# Development and validation of a cuproptosis-related prognostic model for acute myeloid leukemia patients using machine learning with stacking

**DOI:** 10.1038/s41598-024-53306-7

**Published:** 2024-02-02

**Authors:** Xichao Wang, Hao Sun, Yongfei Dong, Jie Huang, Lu Bai, Zaixiang Tang, Songbai Liu, Suning Chen

**Affiliations:** 1 Department of Biostatistics, School of Public Health, Jiangsu Key Laboratory of Preventive and Translational Medicine for Geriatric Diseases, MOE Key Laboratory of Geriatric Diseases and Immunology, Suzhou Medical College of Soochow University, Suzhou, Jiangsu 215123 P. R. China; 2 Suzhou Key Laboratory of Medical Biotechnology, Suzhou Vocational Health College, Suzhou, 215009 Jiangsu China; 3 National Clinical Research Center for Hematologic Diseases, The First Affiliated Hospital of Soochow University, Jiangsu Institute of Hematology, Institute of Blood and Marrow Transplantation, Collaborative Innovation Center of Hematology, Soochow University, Suzhou, China

**Keywords:** Data mining, Machine learning, Statistical methods, Cancer epidemiology, Haematological cancer

## Abstract

Our objective is to develop a prognostic model focused on cuproptosis, aimed at predicting overall survival (OS) outcomes among Acute myeloid leukemia (AML) patients. The model utilized machine learning algorithms incorporating stacking. The GSE37642 dataset was used as the training data, and the GSE12417 and TCGA-LAML cohorts were used as the validation data. Stacking was used to merge the three prediction models, subsequently using a random survival forests algorithm to refit the final model using the stacking linear predictor and clinical factors. The prediction model, featuring stacking linear predictor and clinical factors, achieved AUC values of 0.840, 0.876 and 0.892 at 1, 2 and 3 years within the GSE37642 dataset. In external validation dataset, the corresponding AUCs were 0.741, 0.754 and 0.783. The predictive performance of the model in the external dataset surpasses that of the model simply incorporates all predictors. Additionally, the final model exhibited good calibration accuracy. In conclusion, our findings indicate that the novel prediction model refines the prognostic prediction for AML patients, while the stacking strategy displays potential for model integration.

## Introduction

Acute myeloid leukemia (AML) is a cytogenetically heterogeneous disease. It is defined by abnormal proliferation of progenitor cells in the bone marrow and peripheral blood^1,2^. AML is one of the most common leukemias and has a poor prognosis^3^. According to the National Cancer Institute's SEER (Surveillance, Epidemiology, and End Results) database, from 2014 to 2018, AML accounts for 30% of new leukemia cases, second only to chronic lymphocytic leukemia (36%). The mortality rate for AML was 44.3% among all leukemia subtypes. Chemotherapy is the most conventional treatment for patients with AML. However, cure rates with conventional intensive chemotherapy remain low^4^. With advances in basic medical research, we have gained a better understanding of AML, particularly in terms of the potential mechanisms of AML, environmental and genetic risk factors, and new therapeutic approaches^5^. New therapies (especially targeted therapies and immunotherapy) and new clinical studies are essential to improve the prognosis of AML patients^6^. Predicting the prognostic risk of patients combined with the new understanding is important for advancing clinical treatment.

Cuproptosis is a unique type of cell death because of the accumulation of intracellular copper. This is usually associated with the activity of mitochondria-associated proteins and Fe-S cluster proteins within the cell^7^. Also, leukemia exhibits a high mitochondrial metabolic state^8,9^. More and more studies are focusing on the relationship between cuproptosis and the hematological cancer process. A number of prediction models have been developed to make predictions about specific outcomes in AML patients in current clinical practice^10–12^. However, there are a number of problems with current research on the cuproptosis-related prediction model in hematology. Firstly, the sample size for modeling is insufficient. Secondly, some models have poor predictive performance. Thirdly, some models lack generalization capabilities, especially in research using machine learning algorithms, which frequently experience the problem of overfitting.

The stacking strategy is an alternate option to address these problems. Stacking is a way of combining several low-level prediction algorithms into one high-level prediction algorithm^13^. It is used to combine the advantages of different algorithms and models to improve the prediction performance. In recent years, stacking has been gradually developed and applicated in the medical field^14,15^. However, the application of stacking in AML has not been reported.

Based on the fact, we aimed to construct a prognostic model to predict the overall survival (OS) of AML patients using machine learning algorithms with stacking. We established the cuproptosis-related prognostic model using the GSE37642 dataset and evaluated it in the GSE12417. Then, we used stacking to combine the linear predictors of different models. We constructed the final model with the stacking linear predictor and clinical factors using the random survival forest algorithm. We hope that the novel statistical strategy can provide new insights for future studies.

## Materials and methods

### Data collection and processing

We downloaded RNA-seq data (FPKM values) of 151 patients and corresponding clinical information in TCGA-LAML database from the Genomic Data Commons (GDC) Data Portal (https://portal.gdc.cancer.gov). A total of 136 AML patients with complete survival information were retained.

The GSE37642 and GSE12417 Datasets were downloaded from GEO database (https://www.ncbi.nlm.nih.gov/geo). After excluding samples without complete survival information, 553 and 240 patients were finally included. We adjusted the batch effects using the “normalizeBetweenArrays” function of the “limma” R package^16^.

We downloaded the transcriptome data for the BeatAML cohort from the supplementary file of the article^17^. We also downloaded the clinical and survival information. After integrating the data, 377 patients were eventually included.

### Identifying cuproptosis-related genes

We reviewed 10 cuproptosis-related genes (CRGs) from the literature^7^. We used Spearman rank correlation analysis between CRGs and RNA^18^. To obtain a sufficient number of genes and a low AIC, we used the following selection criteria to indicate RNA related to cuproptosis: Rank correlation coefficients | Rs |> 0.4 and *P* < 0.05 (Table S1).

### Identification and gene ontology (GO) and Kyoto encyclopedia of genes and genomes (KEGG) analysis of overall survival (OS)-related CRGs

The GSE37642 dataset was used to identify CRGs associated with AML survival using univariate Cox hazard regression (*P* < 0.05). We used GO and KEGG analysis to unravel the main functions of OS-related CRGs in AML with the “clusterProfiler” package^19,20^.

### Development and validation of CRGs model

The GSE37642 dataset was used as the training dataset. The validation datasets were the GSE12417 dataset and the TCGA-LAML cohort. We imputed the missing clinical factors using the random forest approach using the “missForest” package^21^.

Among the already identified CRGs, we used Cox hazard regression to identify CRGs associated with the prognosis of AML. The Spike-and-slab Lasso was used for further variable selection with R package “BhGLM”, and we performed tenfold cross-validation with 10 replicates to select an optimal model based on the cross-validated partial log-likelihood (CVPL). It has advantages over Lasso in terms of variable selection and parameter estimation^22,23^. Stepwise regression was conducted using the “stepAIC” function to obtain the optimal gene combinations. Finally, we calculated the following risk scores, and used “cox.zph” function from the “survival” package to test the proportional hazards assumption (*P* > 0.05 was considered to be consistent with the proportional hazards assumption).

$$Risk\, score=\left({\sum }_{i=1}^{n}{\beta }_{i}*{Exp}_{i}\right)$$

where *n* refers to the gene number; ${\beta }_{i}$refers to the coefficient of the gene; and ${Exp}_{i}$ refers to the expression level of the gene.

We evaluated the performance of the model in terms of both discrimination and calibration. Discrimination is the ability of the model to distinguish between patients at different risks^24^. Cumulative/dynamic time-dependent receiver operating characteristic (ROC) curves at 1, 2 and 3 years were plotted using the “survivalROC” package. The area under the ROC curve (AUC) for different years was used to express the discriminatory power of the assessment model over different time scales. The larger values of the AUC provide stronger discriminatory power^25^. We calculated the optimal cut-off value for the linear predictor using the “survminer” package. According to the cut-off value, we divided the patients into two groups with different risks. We used Kaplan–Meier survival analysis to assess the prognostic value of the linear predictor.

Calibration is used to measure the relative difference between the risk of death, specifically the agreement between the predicted risk of death and the observed risk of death^24^. Calibration plots at 1, 2 and 3 years were plotted by the “rms” package to assess the calibration of the Cox hazards model, with a better fit of the curve to the diagonal indicating a better calibration of the model^26^. As for the machine learning model, we calculated the predicted survival probability of each individual at different survival time points. Then, we computed the predicted survival probability for the entire population. We compared the predicted survival probability with the actual survival probability of the population to assess the calibration. The actual survival probability of the population was obtained from the Kaplan–Meier survival analysis.

### Potential relevance of prediction model to therapeutic targets and drug targets

We examined the correlation between cuproptosis-related linear predictors and therapeutic targets using Pearson correlation analysis. Therapeutic targets were systematically reviewed from several previous reports^27–29^. The therapy targets included: ASXL1, BCL2, CD33, CTLA4, CD47, CHEK1, DOT1L, FLT3, IDH1, IDH2, MCL1, MDM2 and PLK1.

We predicted the chemotherapy drugs based on the Cancer Drug Sensitivity Genomics (GDSC) database using the “pRRopheticPredict” function of the “pRRophetic” R package^30^. The Wilcoxon test was used to compare the differences in drug sensitivity between the two groups.

To identify potential drug targets, we analyzed protein-drug interactions within survival-related CRGs using the Drug-Gene Interaction database (DGIdb, https://dgidb.org). Records with an interaction score greater than 1.0 were collected.

### Stacking learning & machine learning

#### Construction of stacking linear predictor

**Figure 1:**
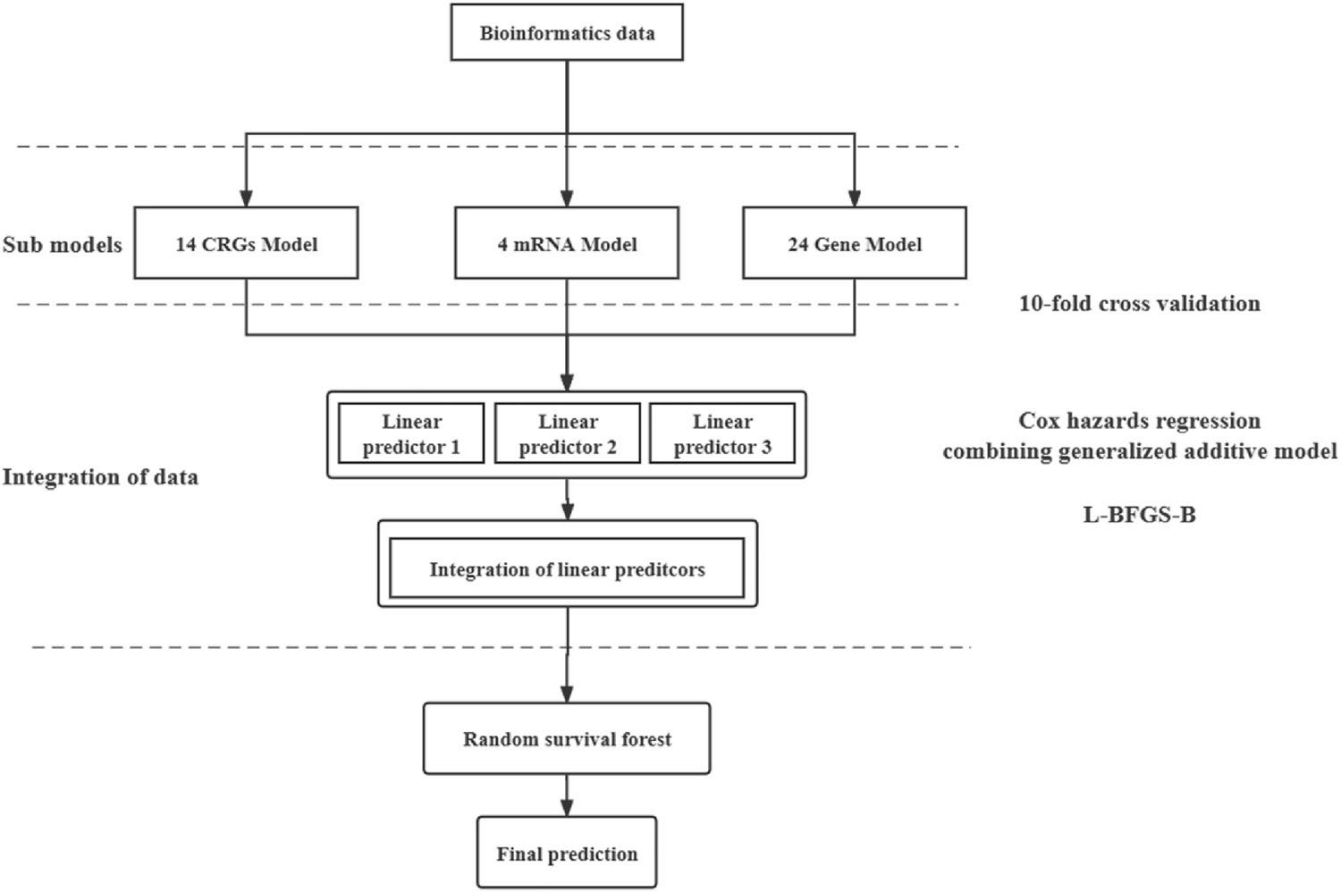
The workflow of stacking. The linear predictors of each sub-model were integrated by tenfold cross-validation using Cox regression combined with a generalized additive model, and the weights of each model were obtained by the L-BFGS-B optimization algorithm.

As is shown in Fig. 1, we first randomly divided the training data (GSE37642 dataset) into 10 equal groups (*n* = 550/10 = 55 observations), which are called “folds”. Secondly, we fit sub-models using nine of the ten folds, calculating the linear predictor in the remaining fold. This process was repeated 10 times, ensuring each fold had a linear predictor. Thirdly, we stacked the linear predictor of each sub-model with the observed outcome of the training data. Fourthly, we fitted the estimates of the models and outcomes with Cox hazards regression combining a generalized additive model. We also imposed a non-negative restriction on the coefficients. Fifthly, we estimated the weights for each sub-model using the limited-memory quasi-Newton method (L-BFGS-B). Finally, we obtained the stacking linear predictor by combining the predicted values of the sub-models with their corresponding weights.

#### Machine learning algorithms

We utilized four machine learning algorithms to develop a survival prediction model, including random survival forest (RSF), survival support vector machine (survival-SVM), generalized boosted regression modeling (GBM), and eXtreme Gradient Boosting (XGBoost). The RSF is a random forest method for the analysis of right-censored survival data^31^. This method uses the “randomForestSRC” R package. The survival-SVM is an approach based on SVM, which searches through the utility functions of covariates to obtain utility values that are as consistent as possible with the corresponding observed failures^32^. The GBM is a nonparametric method for building a collection of decision tree sequences. It iteratively increases the basis functions so that each additional basis function further reduces the chosen loss function^33^. The XGBoost is a tree-based approach that handles unscaled data. tenfold cross-validation with Grid search was used on the whole dataset for hyperparameter tuning to identify the optimal configurations^34^. The hyperparameters tuned, ranges and final configurations for these machine learning models are available in Table S2.

We employed these algorithms to construct the model, utilizing the stacking linear predictor and clinical factors. Subsequently, the crude performance of these four models is used to select the optimal machine learning model for building the final model. Internal validation was carried out by a 1000-times bootstrap method.

#### Improvement of ELN recommendation

We redefined the risk groupings for AML by integrating the ELN2017 groups, along with the new risk groups distinguished by the RSF model. Then we compared differences in risk among the groups.

### Statistical analysis

All statistical analyses were performed using R (version 4.1.0). Student’s t-test or Mann–Whitney test was used to determine the relationship between the linear predictor and clinical factors. *P* < 0.05 was considered statistically significant. Survival curves were analyzed using the log-rank test. Bonferroni correction was used to control for Type I errors.

## Results

### Participant characteristics

Demographic and clinical characteristics of these populations are detailed in Table 1. After adjusting for batch effects, the genetic data in the three datasets are comparable.

**Table 1:** Distribution of variables in the populations.

Variables	GSE37642 (*N* = 553)	GSE12417 (*N* = 240)	TCGA-LAML (*N* = 131)
Median survival time (day)	353	396	577
Age (Mean ± SD)	54.90 ± 14.81	56.98 ± 14.72	53.46 ± 16.25
Status, *n* (%)			
Dead	406 (73.4)	150 (62.5)	80 (61.1)
Alive	127 (26.6)	90 (37.5)	51 (38.9)
FAB, *n* (%)			
0	22 (4.1)	6 (2.5)	12 (9.2)
1	113 (21.0)	68 (28.3)	32 (24.4)
2	164 (30.5)	79 (32.9)	32 (24.4)
3	26 (4.8)	0 (0)	13 (9.9)
4	121 (22.5)	53 (22.1)	27 (20.6)
5	66 (12.3)	25 (10.4)	12 (9.2)
6	22 (4.1)	9 (3.8)	2 (1.5)
7	3 (0.6)	–	1 (0.8)
Missing	16 (2.9)	–	–
Runx1 mutation, *n* (%)			
Yes	75 (15.2)	–	–
No	419 (84.8)	–	–
Missing	59 (10.7)	–	–
Runx1.runx1t1 fusion, *n* (%)			
Yes	30 (5.4)	–	–
No	523 (94.6)	–	–
ELN2017, *n* (%)			
1	–	–	29 (22.5)
2	–	–	73 (56.6)
3	–	–	27 (20.9)
Missing	–	–	2 (1.5)
ARPC5L (Mean ± SD)	10.12 ± 0.51	10.03 ± 0.54	8.13 ± 0.51
CYP19A1 (Mean ± SD)	5.61 ± 0.27	5.81 ± 0.25	5.07 ± 0.30
ESYT1 (Mean ± SD)	9.06 ± 0.64	8.98 ± 0.56	10.93 ± 0.53
FDXR (Mean ± SD)	6.15 ± 0.50	6.18 ± 0.50	6.85 ± 0.42
HSPD1 (Mean ± SD)	11.61 ± 0.59	11.57 ± 0.55	10.29 ± 0.64
IGLL1 (Mean ± SD)	7.23 ± 1.59	7.56 ± 1.69	9.47 ± 2.21
KRBOX4 (Mean ± SD)	6.77 ± 0.61	6.75 ± 0.89	7.30 ± 0.25
PLPP3 (Mean ± SD)	7.14 ± 0.81	7.09 ± 0.78	5.93 ± 0.38
RIOK2 (Mean ± SD)	7.68 ± 0.47	7.58 ± 0.54	7.16 ± 0.28
STK25 (Mean ± SD)	7.72 ± 0.46	7.60 ± 0.53	8.37 ± 0.48
TNKS2 (Mean ± SD)	9.15 ± 0.54	9.09 ± 0.67	9.56 ± 0.41
TRIM8 (Mean ± SD)	8.96 ± 0.65	8.96 ± 0.66	9.25 ± 0.77
ULK1 (Mean ± SD)	8.20 ± 0.53	8.23 ± 0.48	8.82 ± 0.74
ZMIZ1 (Mean ± SD)	10.47 ± 0.60	10.51 ± 0.54	10.04 ± 0.86

### Identification and functional enrichment analysis of OS-related CRGs in AML

**Figure 2:**
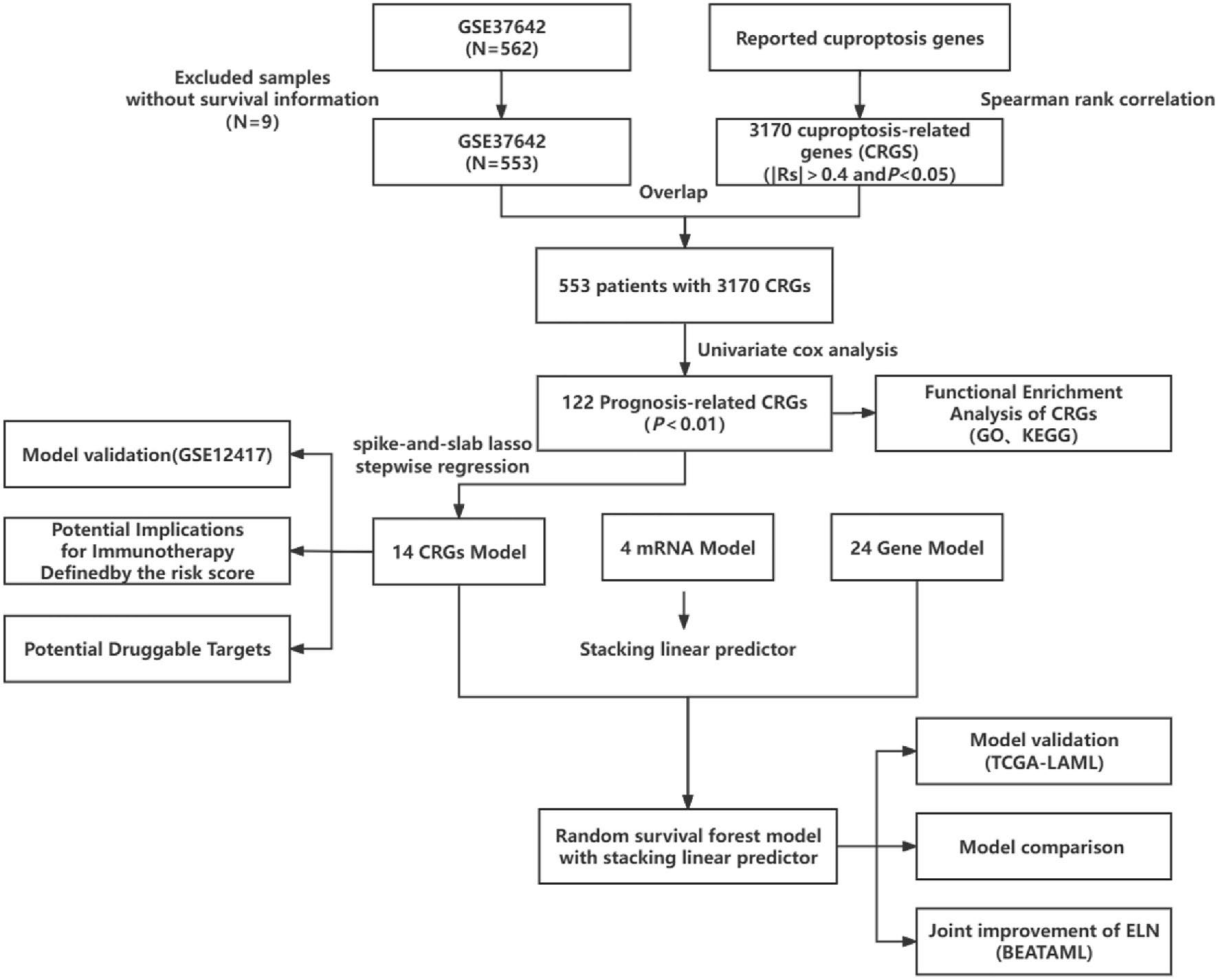
The workflow of this study.

The workflow for this study is illustrated in Fig. 2. The reviewed CRGs are listed in Table S3. A list of the 3170 genes obtained through Spearman correlation is provided in Table S4. We identified 122 copper death-related genes associated with AML prognosis by univariate Cox hazard regression (Table S5). KEGG results indicated the activity of these CRGs in the process of phagocytosis and mRNA surveillance pathway (Figure S1A). Additionally, these genes were involved in the process of macroautophagy and ‘De novo’ protein folding (Figure S1B).

### Development and validation of the cuproptosis-related risk score

In the dataset of GSE37642, we screened and obtained 22 genes from the 122 OS-related CRGs using the Spike-and-slab lasso method (Figure S2). Then, we obtained a risk score containing 14 OS-Related CRGs genes through stepwise regression. The risk score is presented below:

$$Risk score=0.5170\times ARPC5L+0.7593\times CYP19A1-0.3070\times ESYT1-0.2311\times FDXR+0.3795\times HSPD1-0.0791\times IGLL1+0.3644\times KRBOX4+0.2472\times PLPP3-0.4239\times RIOK2+0.4301\times STK25-0.3409\times TNKS2+0.2820\times TRIM8-0.3810\times ULK1+0.2613\times ZMIZ1$$

**Figure 3:**
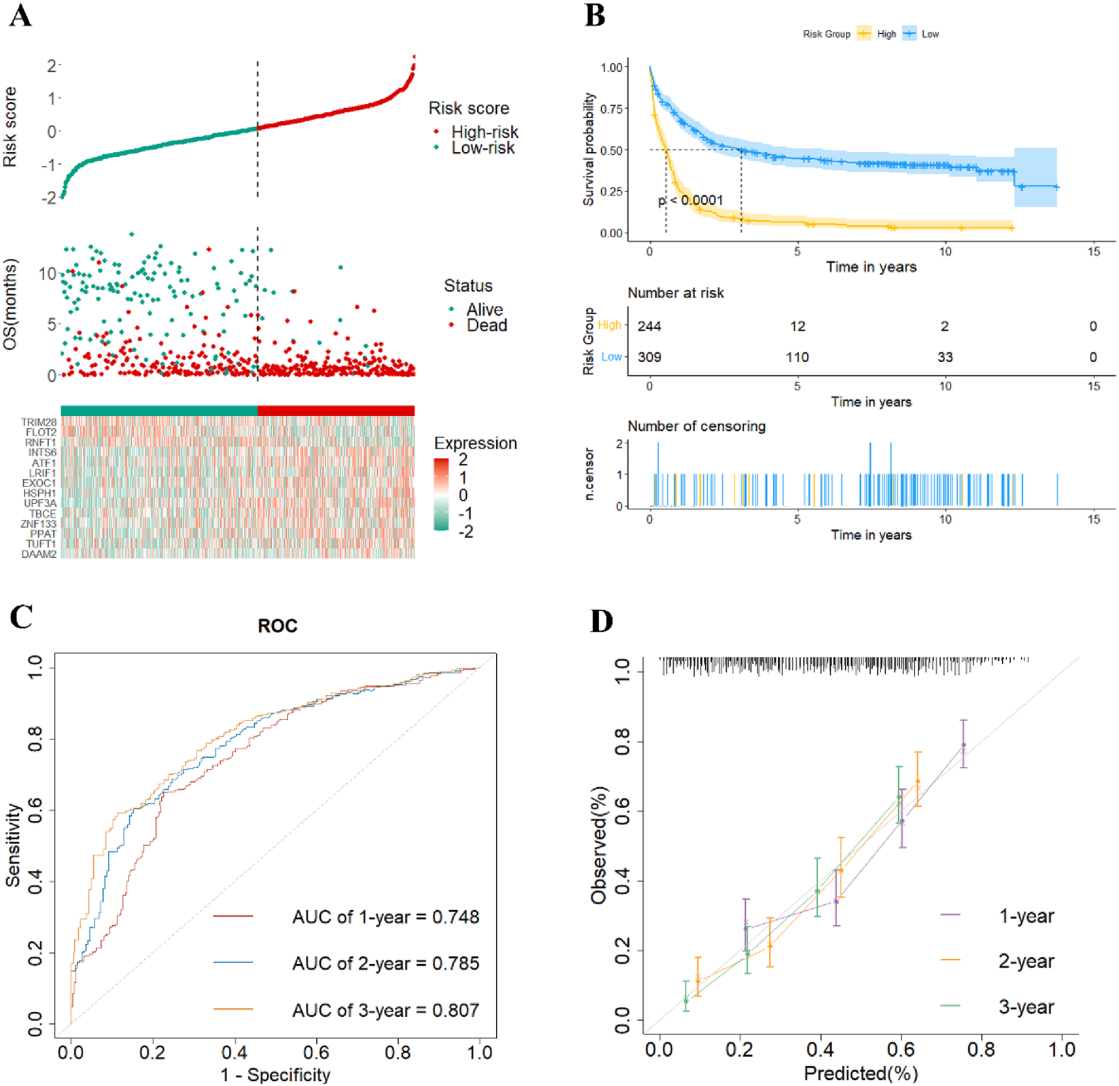
Identification of a risk signature for OS in the GSE37642 dataset. (**A**) The optimal cutoff value of risk score; (**B**) The Kaplan–Meier plot shows patient OS differences based on risk score stratification; (**C**) The 1, 2 and 3 years ROC curves; (**D**) The calibration plot.

The time-dependent ROC at 1, 2 and 3 years demonstrated the good discriminative power of the risk score across different time horizons (Fig. 3C). Patients were divided into high-risk groups and low-risk groups based on the cut-off value of the risk score (0.08), revealing significant differences in the two survival curves (Fig. 3A,B). The calibration plots (Fig. 3D) indicated the good calibration accuracy of the model.

**Figure 4:**
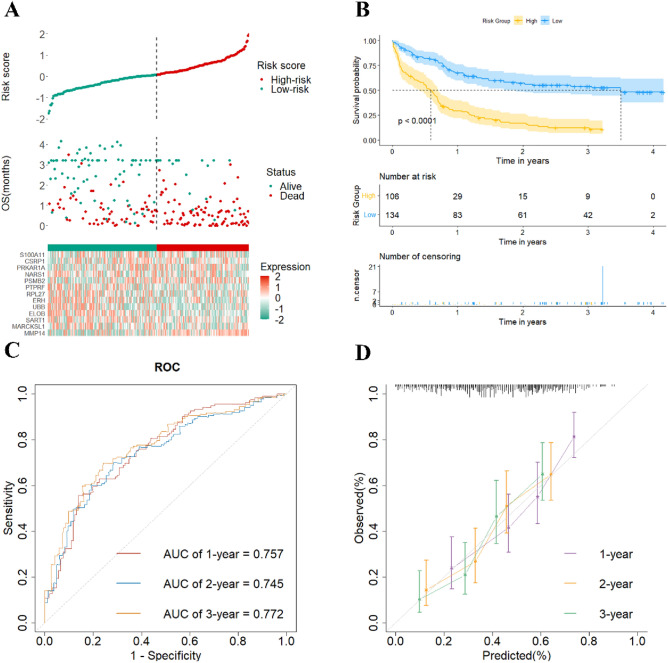
Validation of a risk signature for OS in the GSE12417 dataset. (**A**) The optimal cutoff value of risk score; (**B**) The Kaplan–Meier plot shows patient OS differences based on risk score stratification; (**C**) The 1, 2 and 3 years ROC curves; (**D**) The calibration plot.

In the validation dataset of GSE12417, the time-dependent ROC curves (Fig. 4C), the survival curves of the two risk groups (Fig. 4A,B), and the calibration plots (Fig. 4D) all demonstrated strong performance of prediction.

We categorized the patients into two groups by age, specifically those below and above 60 years old. Across different age groups, the CRGs model consistently exhibited the ability to classify patients into high and low risk groups, with *p*-values for the log-rank test being less than 0.05 (Figure S3,S4).

### Potential relevance of risk score in tumor-immune microenvironment

**Figure 5:**
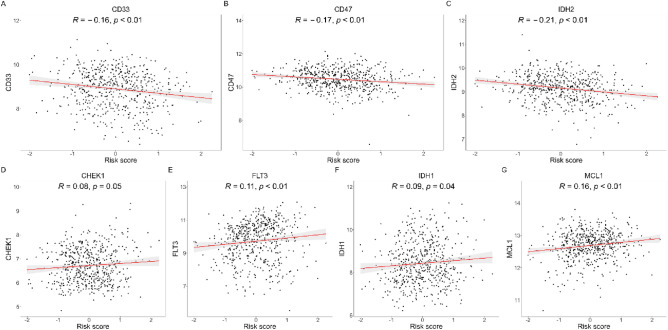
Pearson correlation of the risk scores of the targets of immunotherapy and targeted therapy. (**A**-**C**) The negative correlations between the risk score and the mRNA expression levels of CD33, CD47, and IDH2; (**D**-**G**) The positive correlations between the risk score and the mRNA expression levels of CHEK1, FLT3, IDH1, and MCL1.

Pearson correlation analysis (Fig. 5) revealed negative correlations between the risk score and the mRNA expression levels of CD33 (R =  − 0.16, *P* < 0.01), CD47 (R =  − 0.17, *P* < 0.01), IDH2 (R =  − 0.21, *P* < 0.01). Conversely, the risk score was positively related to the mRNA expression levels of CHEK1 (R = 0.08, P = 0.05), FLT3 (R = 0.11, *P* < 0.01), IDH1 (R = 0.09, P = 0.04), MCL1 (R = 0.16, *P* < 0.01). IC50 was calculated for each AML patient in the two different risk groups. We plotted the top 10 different significantly different treatment-sensitive drugs (Figure S5). Risk scores could be used to predict sensitivity to these drugs for AML patients. Using the DGIbd database, we identified 18 CRGs as targets among the 50 predicted drugs (Table 2).

**Table 2:** 18 OS-related CRGs targeted by the drugs by DGIdb.

Gene	HR	95%CI	*P*-value	Number of drugs	Drug name
BAP1	0.61	0.45–0.83	< 0.001	2	OLAPARIB, PANOBINOSTAT
CDKN2A	0.77	0.63–0.93	0.01	5	MILCICLIB MALEATE, HMN-214, GSK-461364, PALBOCICLIB, ABEMACICLIB
CYP19A1	1.82	1.27–2.61	< 0.001	10	ANASTROZOLE, TESTOLACTONE, EXEMESTANE, LETROZOLE, CHEMBL1077603, CHEMBL572637, AMINOGLUTETHIM, ATAMESTANE, ARIMIDEX, ISOPROPYL ALCOHOL
HSPA9	0.78	0.64–0.94	0.01	1	CHEMBL33859
HSPD1	1.35	1.12–1.61	< 0.001	2	CETRORELIX, DIAPEP-277
IDH2	0.78	0.66–0.93	0.01	4	ENASIDENIB, VENETOCLAX, SARACATINIB, QUIZARTINIB
ITGA4	0.82	0.71–0.93	< 0.001	6	NATALIZUMAB, VEDOLIZUMAB, CHEMBL88478, FIRATEGRAST, ABRILUMAB, SENKTIDE
KCNJ2	1.2	1.06–1.36	0.01	1	DRONEDARONE HYDROCHLORIDE
MME	1.21	1.05–1.39	0.01	8	CANDOXATRIL, LCZ696, SAMPATRILAT, SLV-334, SACUBITRIL, PEPINEMAB, ILEPATRIL, GALLOPAMIL
MPST	0.75	0.61–0.91	< 0.001	1	THYROXINE
PDCD4	0.74	0.59–0.92	0.01	1	PACLITAXEL
PLCG2	1.3	1.07–1.57	0.01	1	IBRUTINIB
PPAT	1.4	1.16–1.69	< 0.001	3	AZATHIOPRINE SODIUM, MERCAPTOPURINE, AZATHIOPRINE
PPP2CA	0.73	0.61–0.87	< 0.001	1	LB-100
PSMD3	0.81	0.69–0.95	0.01	1	CARFILZOMIB
SLC39A14	1.21	1.07–1.36	< 0.001	1	NORTRIPTYLINE
TBXAS1	0.85	0.76–0.95	< 0.001	1	PYRIDINE
VCP	0.74	0.61–0.9	< 0.001	1	CB-5083

18 survival-related CRGs are selected by the Drug-Gene Interaction database with an interaction score greater than 1.0.

### Construction of stacking linear predictor

Predictors from an international collaborative study^35^ and a study validated by multiple independent cohorts^36^ were reviewed to construct the stacking linear predictor. Through tenfold cross-validation, we obtained the weights of the sub-linear predictors (CRGs: 0.68; 4-mRNA model: 0.25; 24-Gene model: 0.07). The stacking linear predictor is constructed by the following equation:

$$Stacking lp=0.68\times {CRG}{\prime}s lp+0.25\times {4mRNA model}{\prime}s lp+0.07\times {24gene model}{\prime}s lp$$

where *lp* refers to the linear predictor of the model.

**Figure 6:**
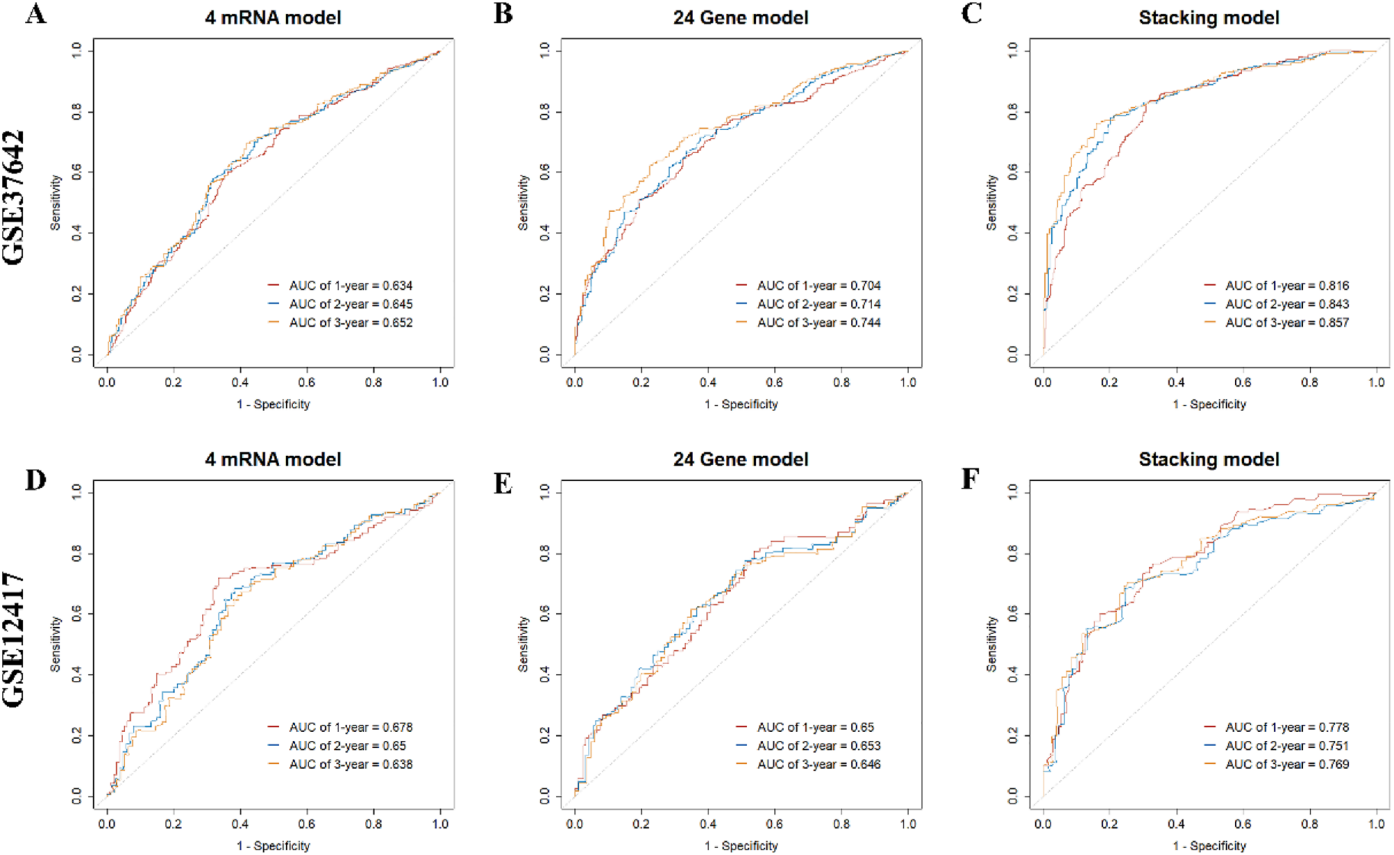
Comparison of 1-, 2- and 3-year ROC curves for different sub-models and stacking model. (**A**, **B**): The 1, 2 and 3 years ROC curves of sub models in GSE37642; (**C**): The 1, 2 and 3 years ROC curves of the stacking model in GSE37642; (**D**, **E**): The 1, 2 and 3 years ROC curves of sub-models in GSE12417; (**F**): The 1, 2 and 3 years ROC curves of the stacking model in GSE12417.

We compared the predictive abilities of sub-models and stacking linear predictors. We observed an improved predictive ability of stacking linear predictors compared to the sub-models (Fig. 6). The stacking model was evaluated using a 1000-times bootstrap method and showed high discrimination with an average AUC of 0.810 (95% CI: 0.773–0.847) (Figure S6). The model with stacking linear predictor performs better in the external validation dataset than the model with a simple combination of all predictors. (Figure S7).

### Development and validation of the RSF model

**Figure 7:**
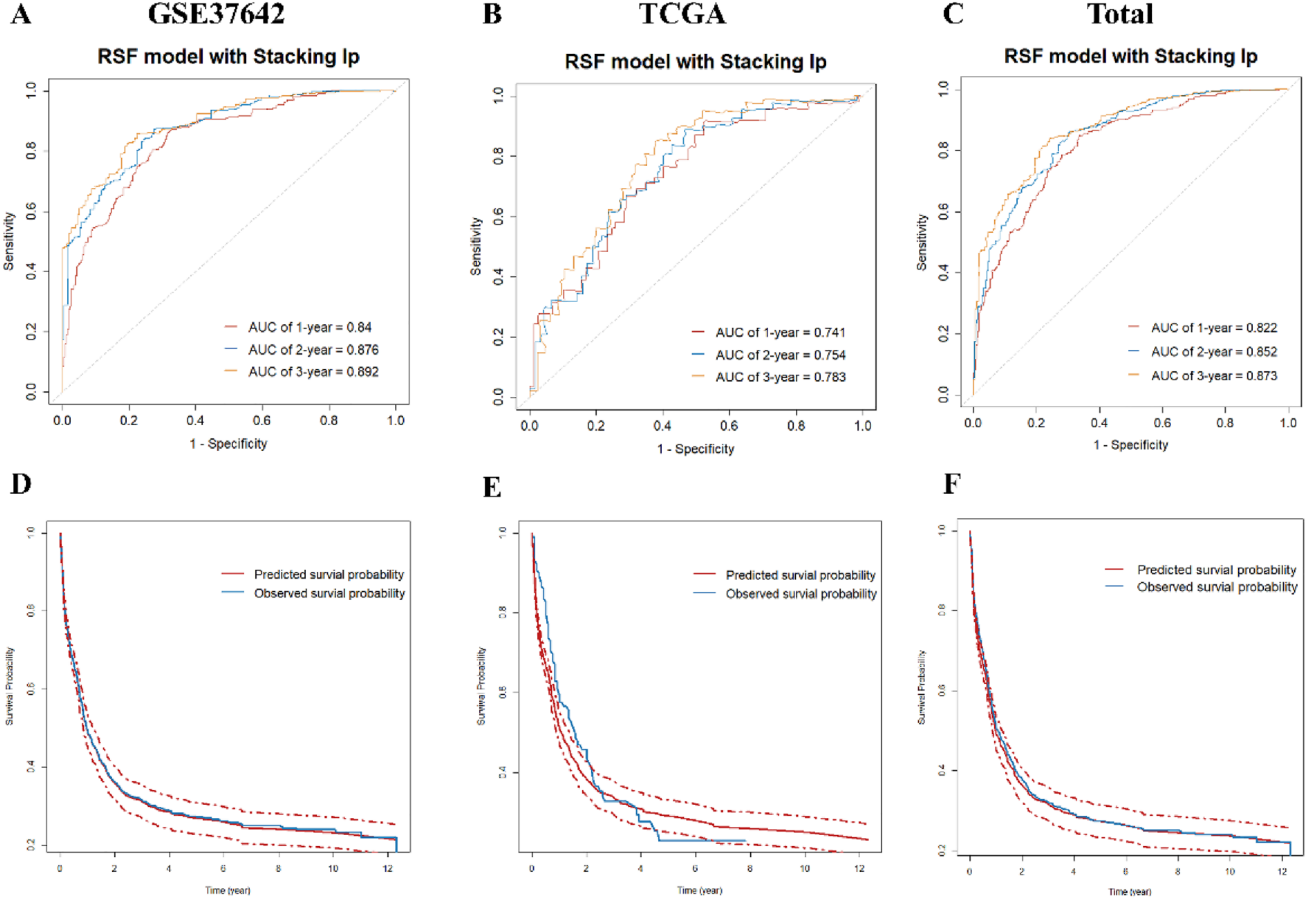
Identification and validation of RSF model with stacking linear predictor. (**A**) The 1, 2 and 3 years ROC curves of RSF model in training dataset (GSE37642); (**B**, **C**) The 1, 2 and 3 years ROC curves of RSF model in validation dataset (TCGA and merged data); (**D**) The calibration plot of RSF model in training dataset; (**E**, **F**) The calibration plot of RSF model in validation dataset.

We constructed a multivariate Cox hazard regression analysis using the established stacking linear predictor along with certain clinical factors to investigate whether the predictor could be used as an independent prognostic factor. In the GSE37642 dataset, the stacking linear predictor emerged as an independent prognosis predictor for AML patients after adjusting for age and FAB stage (P < 0.001). The comprehensive model was constructed using the obtained stacking linear predictor, age, and FAB stage, using each of the four machine learning methods (RSF, Survival-SVM, GBM, and XGBoost). Figure S8 shows the time-dependent AUC of stacking final models based on these four machine learning methods, and it turns out that the RSF algorithm has the optimal model discrimination. Therefore, we used the RSF-based stacking model to construct a prediction model for AML patients. The 1, 2 and 3 years AUCs of the model were 0.840, 0.876 and 0.892 (Fig. 7A). 1000-times bootstrap method showed mean AUC = 0.878 (95% CI: 0.848–0.908) (Figure S9). In the external validation dataset (TCGA), the model achieved AUC values of 0.741, 0.754 and 0.786 at the 1, 2 and 3 years, respectively (Fig. 7B). As shown in Fig. 7D,E, the calibration of the model is still acceptable. We merged the two datasets and validated the model in the merged dataset. The result is still stable in this dataset (Fig. 7C,F).Figure S10 shows dead probabilities prediction plots generated using this prediction model for 3 patients. Three patients had different probabilities of death at different times, suggesting that the prediction model may have moderate differences in predicting survival for patients.

**Figure 8:**
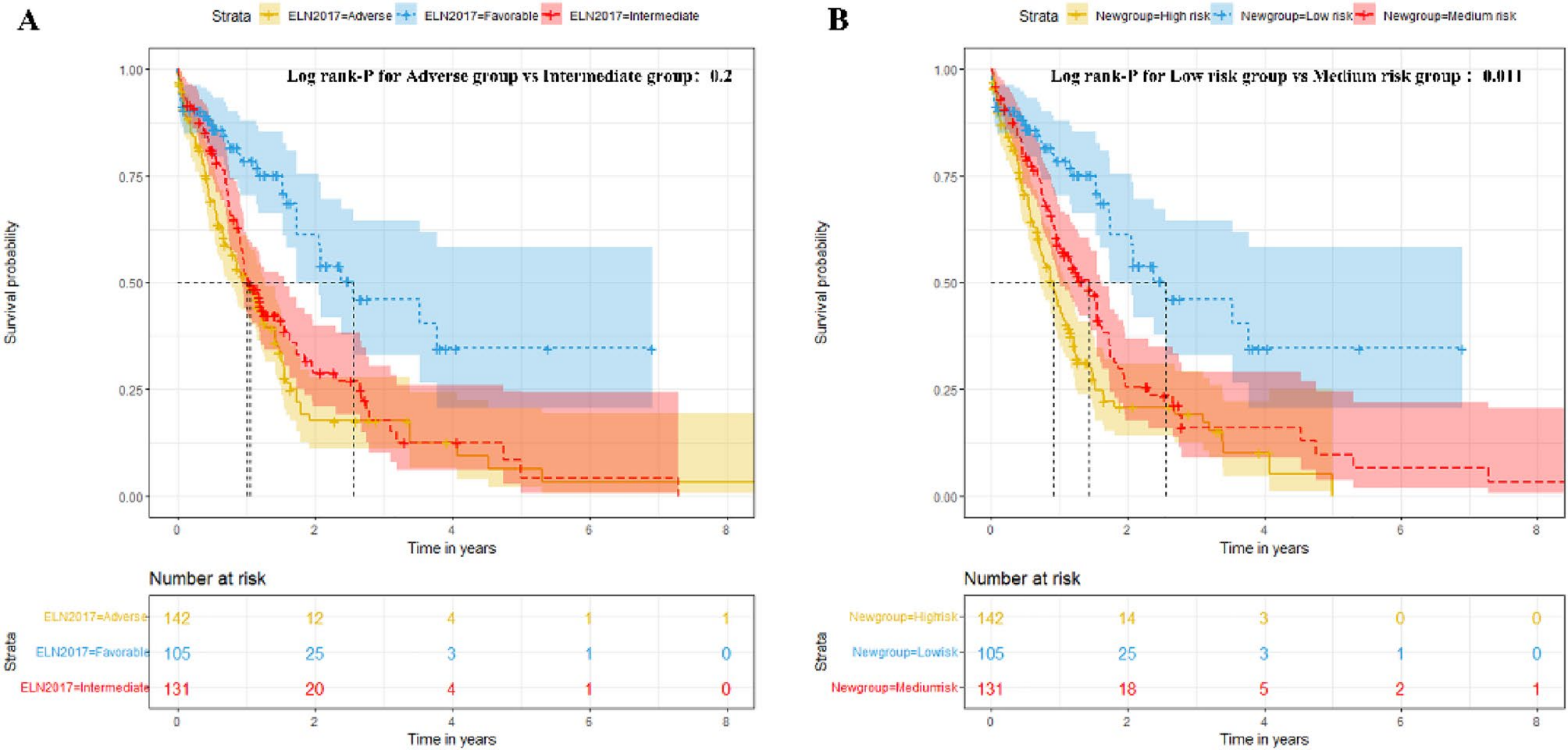
The Kaplan–Meier plot. **(A)** The Kaplan–Meier plot shows patient OS differences based on ELN stratification; **(B)** The Kaplan–Meier plot shows patient OS differences based on new stratification; Bonferroni-corrected *P* < 0.017 was considered statistically significant.

To further validate the predictive power of the model, we assessed the combined effect with ELN2017 risk stratification in the BeatAML cohort. In the BeatAML cohort, AML patients were divided into “Favorable”, “Intermediate” and “Adverse” groups according to ELN2017 criteria. We plotted the survival curves for these three groups, the separation between the “Intermediate” and “Adverse” groups was not clear (the P value of the log-rank test is 0.2; Fig. 8A). Subsequently, patients in the BeatAML cohort were classified into new risk groups based on the RSF model. We merged the ELN2017 grouping criteria and the RSF model's grouping criteria: The ELN Favorable group is considered a low-risk group, the ELN “Intermediate” group and the RSF low-risk group or ELN “Adverse” group and the RSF low-risk groups are considered an intermediate group and the remaining three subgroups are a high-risk group. We additionally plotted the survival curves for these new three groups. The P value of the log-rank test between the three groups was less than 0.01, and the P value of the log-rank test between the medium-risk group and high-risk group was 0.011 (Fig. 8B).

## Discussion

We identified 14 cuproptosis-related genes (CRGs) associated with OS in AML patients. We reviewed these 14 CRGs in the context of AML progression. The study on the epigenetic and genetic heterogeneity of AML showed that high expression of IGLL1 is associated with cell cycle and DNA repair^37^. RIOK2 has been reported as a potential therapeutic target for AML. Deletion of RIOK2 leads to reduced protein synthesis and ribosomal instability, leading to apoptosis in leukemia cells^38^. At the same time, RIOK2 inhibition targets protein synthesis instead of targeting the PI3K/AKT/mTOR pathway, a pathway that is implicated in the mechanism of AML publication. This suggests potential clinical validity in AML therapy^38,39^. High expression of STK25 has been reported to be associated with poor prognosis in AML patients, and the silence of STK25 promotes AraC-induced apoptosis and inhibits AML cell proliferation^40^. ULK1 has been reported as a potential therapeutic target for AML, and ULK1 itself is a key gene associated with autophagy^41^. ULK1 inhibitors effectively induce mitochondria-mediated, caspase-dependent apoptosis in FLT3-ITD-mutated leukemia cell lines and primary leukemia cells^42^. We also reviewed these 14 CRGs in the context of cell death. FDXR is essential for the biogenesis of iron-sulfur (Fe-S) clusters, and the instability of Fe-S clusters may further contribute to the onset of cuproptosis^43^. HSPD1 has also been reported to be associated with cuproptosis^44^. TRIM8 has emerged as a crucial regulator of cell survival, apoptosis, and oxidative stress in various pathological processes^45^. However, no study has reported a correlation between TRIM8 and cuproptosis. Other genes have also been confirmed to be strongly associated with leukemia or other diseases^46–49^. We did not find any report on KRBOX4. In aggregate, these studies indicate the potential significance of biomarkers in the context of cuproptosis and AML progression.

Intensive chemotherapy is currently the main treatment for AML patients, but it is not suitable for all individuals due to age and other comorbidities. Targeted therapies offer new treatment strategies. The CRGs risk score was negatively correlated with CD33, CD47, and IDH2, and positively correlated with CHEK1, FLT3, IDH1, and MCL1. This suggests that patients may not respond well to the former inhibitors but may benefit from blocking CHEK1, FLT3, IDH1, and MCL1. CHEK1 plays a crucial role in the DNA damage response by halting the cell cycle and allowing ample time for DNA repair. In AML, where DNA damage is frequent, CHEK1 abnormalities may lead to an inadequate cellular response to DNA damage, increasing AML cell survival and proliferation^50^. FLT3 mutations may cause aberrant activation of the FLT3 signaling pathway, contributing to the abnormal proliferation of AML cells. FLT3 inhibitors induce apoptosis and increase the sensitivity of AML cells to other drugs^51^. Mutated IDH1 is relatively common in AML, especially the R132H mutation, which can alter metabolic pathways and affect cell differentiation, creating an environment for AML to occur^52^. In AML, MCL1 is commonly overexpressed and helps maintain AML cell survival. Strategies to inhibit MCL1 are thought to be a way to treat AML by driving AML cells into apoptosis^53^.

To date, there has been a proliferation of articles focusing on the utilization of omics data^29^. In addition, machine learning and deep learning methods are gaining popularity among researchers in the field of medicine^54,55^. On the one hand, machine learning algorithms are employed for feature extraction^56^. On the other hand, machine learning and deep learning algorithms are directly applied for classification or survival analysis^57^. However, machine learning tends to overfit training data, possibly capturing localized answers within a particular patient sample or a small group of samples, which may not generalize to broader patient populations^58^. To address the problem, we used stacking to combine three prediction models and computed the stacking linear predictor. Then, we used the machine learning method to fit the final model, incorporating the stacking linear predictor and clinical factors. The model developed in this study exhibits superior effectiveness compared to the sub-models. Moreover, the stability of the machine learning model featuring stacking linear predictors was confirmed in external validation dataset.

The model in this study outperforms previous models in terms of predictive performance. Unlike other previous models, which lacked sufficient validation, we fully validated our model, including internal validation using the bootstrap method and external validation, demonstrating its generalization capability. The bootstrap method also helps to address the problem of biased estimation under small samples. Meanwhile, we fully considered the model evaluation metrics. The corresponding calibration curves for the machine learning methods are plotted.

The primary highlight of this paper is the enrichment focus on the cuproptosis study within AML, coupled with the standardized model construction process. The second highlight of this paper is the combination of machine learning with stacking, which facilitates the combination of multiple omics data and multiple existing models. Most importantly, this strategy effectively mitigates the challenge of overfitting. Several limitations pertain to this study. Firstly, the datasets employed here may still have the potential heterogeneity after being adjusted and had limited availability of common clinical factors. However, we demonstrated the stability and generalizability of our findings through sensitivity analyses in datasets with potential heterogeneity. Secondly, despite the existence of ELN2022 recommendations, we opted for the ELN2017 grouping standard due to the absence of an AML dataset containing sufficient genetic data to define ELN2022 groups. However, our study provided a new strategy for integrating risk scores and validated the feasibility of the strategy at different dimensions. The lack of clinical data limits further studies of the correlation between the integrating risk score and clinical factors.

In conclusion, our study used stacking and machine learning to establish a prognostic model for OS prediction in AML patients. The model exhibited superior performance in external datasets compared to machine learning models that directly incorporate predictors. Furthermore, the model offers novel insights into potential risk stratification and treatment strategies. It is our anticipation that the insights garnered from our investigation into cuproptosis within AML prognosis, facilitated by statistical research strategies, will enhance the diagnosis of AML, drive innovations in treatment approaches, and contribute to the extension of patient survival.

### Supplementary Information

Supplementary Information 1.

Supplementary Tables.

## Data Availability

Raw microarray datasets of GSE37642 and GSE12417 were downloaded from GEO database. (https://www.ncbi.nlm.nih.gov/geo/query/acc.cgi?acc=GSE37642, https://www.ncbi.nlm.nih.gov/geo/query/acc.cgi?acc=GSE12417). RNA-seq data and corresponding clinical information in TCGA-LAML database were downloaded from the Genomic Data Commons Data Portal (https://portal.gdc.cancer.gov/repository). The transcriptome data and clinical information for the BeatAML cohort during this study are included in the published article (and its Supplementary Information files). (Functional genomic landscape of acute myeloid leukaemia | Nature).
