# Supplementary material for: Development and validation of a cuproptosis-related prognostic model for acute myeloid leukemia patients using machine learning with stacking
Source: Sci Rep. 2024 Feb 2;14:2802. doi: 10.1038/s41598-024-53306-7 (PMC10837443; doi:10.1038/s41598-024-53306-7)

Development and validation of a cuproptosis-related prognostic model for acute myeloid leukemia patients using machine learning with stacking

Xichao Wang, et al.

Supplementary materials

Outline

[Materials and methods 4](#_Toc155216024)

[The consideration of different correlation coefficients. 4](#_Toc155216025)

[Table S1 Number of genes and AIC under different criteria. 4](#_Toc155216026)

[GO and KEGG Analysis of Overall Survival-Related CRGs 5](#_Toc155216027)

[Figure S1 Significantly enriched GO terms and KEGG pathways of OS-related CRGs. (A) Significantly enriched pathways of OS-related CRGs, (B) Significantly enriched GO terms of OS-related CRGs. 5](#_Toc155216028)

[Cuproptosis-related risk score development and validation. 6](#_Toc155216029)

[Figure S2 The solution paths and coefficients of the spike-and-slab lasso Cox model. (A) The solution path for spike-and-slab lasso Cox model. (B) Spike-and-slab lasso coefficients plot. 6](#_Toc155216030)

[Figure S3 The Kaplan-Meier plot of different age groups in GSE37642 dataset. (A) The Kaplan-Meier plot shows OS differences of those age ≤60 years according to risk score stratification. (B) The Kaplan-Meier plot shows OS differences of those age >60 years according to risk score stratification. 7](#_Toc155216031)

[Figure S4 The Kaplan-Meier plot of different age groups in GSE12417 dataset. (A) The Kaplan-Meier plot shows OS differences of those age ≤60 years according to risk score stratification. (B) The Kaplan-Meier plot shows OS differences of those age >60 years according to risk score stratification. 8](#_Toc155216032)

[Stacking model validation and comparisons for different machine learning 9](#_Toc155216033)

[Table S2 Setting of hyper-parameters for different machine learning. 9](#_Toc155216034)

[Figure S5 The discrepancies of top 10 drug sensitivity by p value between different risk groups. Smaller IC50 values indicate greater sensitivity to the drug. 10](#_Toc155216035)

[Figure S6 Bootstrap ROC for stacking model. Internal validation was carried out by a 1000-times bootstrap method. 11](#_Toc155216036)

[Figure S7 Comparison of 1-,2-,3-year ROC curves for random survival forest model with different predictors in validation dataset (TCGA-LAML). (A) Random survival forest model with stacking linear predictor; (B) Random survival forest model simply incorporates all predictors. 12](#_Toc155216037)

[Figure S8 Comparison of time dependent AUC among different machine learning. 13](#_Toc155216038)

[Figure S9 Bootstrap ROC for RSF model with stacking linear predictor. Internal validation was carried out by a 1000-times bootstrap method. 14](#_Toc155216039)

[Personalised Prediction 15](#_Toc155216040)

[Figure S10 Individual predictions for AML patients using rsf model with stacking linear predictor and clinical factors. 15](#_Toc155216041)

## Materials and methods

### The consideration of different correlation coefficients.

In order to determine the specific Rs values, we made the following considerations: firstly, as the first step of dimensionality reduction, too many genes do not serve the purpose of dimensionality reduction; secondly, we don't want to lose too much amount of information on the basis of searching for the genes related to the specific cuproptosis-related factors, so too small number of genes is not the reasonable result. Thirdly, in order to evaluate the results with different Rs values, we measured our results with the AIC of spike and slab lasso. (**Table S1**).

### Table S1 Number of genes and AIC under different criteria.

| Rs | Number of genes | AIC |
| --- | --- | --- |
| 0.3 | 6598 | 4553.14 |
| 0.4 | 3208 | 4522.06 |
| 0.5 | 1103 | 4590.69 |
| 0.6 | 169 | 4522.51 |

Rs = Rank correlation coefficients;

AIC = Akaike information criterion.

## GO and KEGG Analysis of Overall Survival-Related CRGs

### Figure S1 Significantly enriched GO terms and KEGG pathways of OS-related CRGs. (A) Significantly enriched pathways of OS-related CRGs, (B) Significantly enriched GO terms of OS-related CRGs.


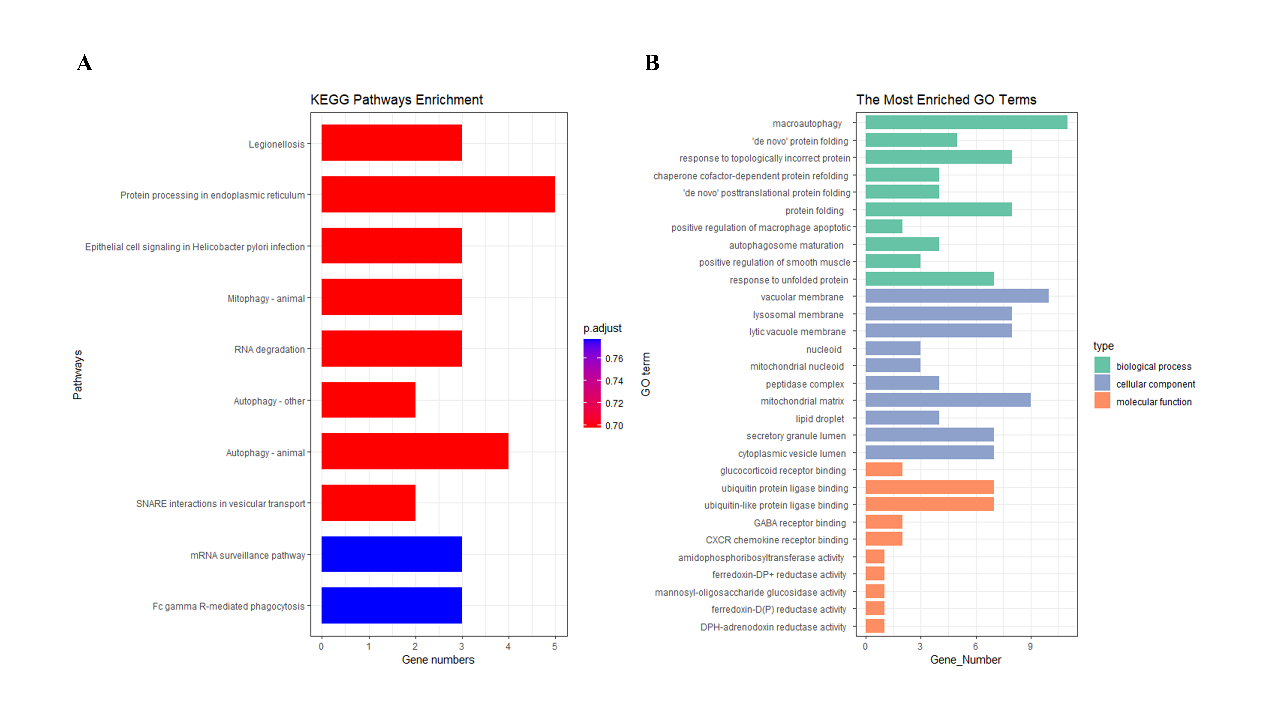


## Cuproptosis-related risk score development and validation.

### Figure S2 The solution paths and coefficients of the spike-and-slab lasso Cox model. (A) The solution path for spike-and-slab lasso Cox model. (B) Spike-and-slab lasso coefficients plot.


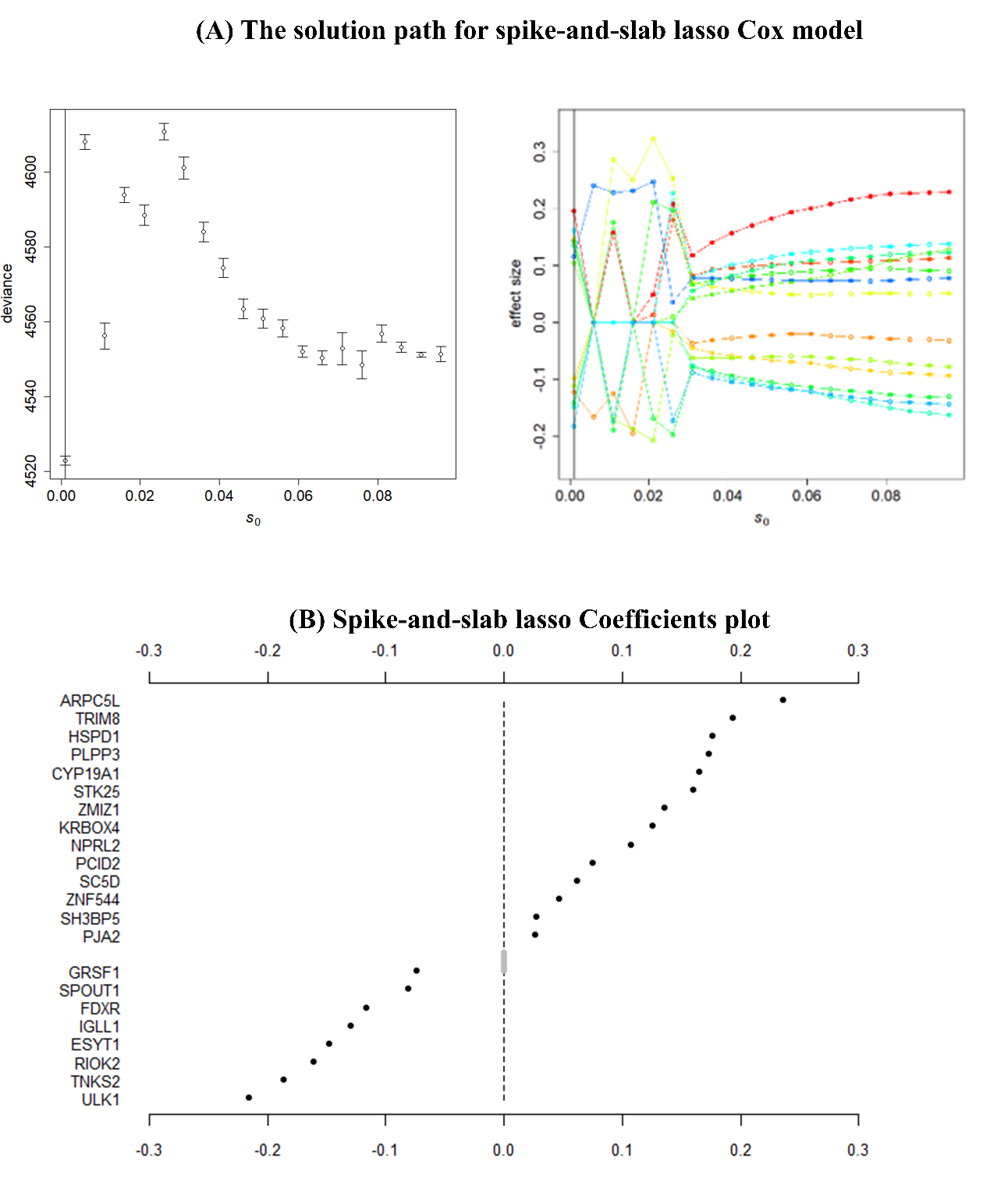


### Figure S3 The Kaplan-Meier plot of different age groups in GSE37642 dataset. (A) The Kaplan-Meier plot shows OS differences of those age ≤60 years according to risk score stratification. (B) The Kaplan-Meier plot shows OS differences of those age >60 years according to risk score stratification.


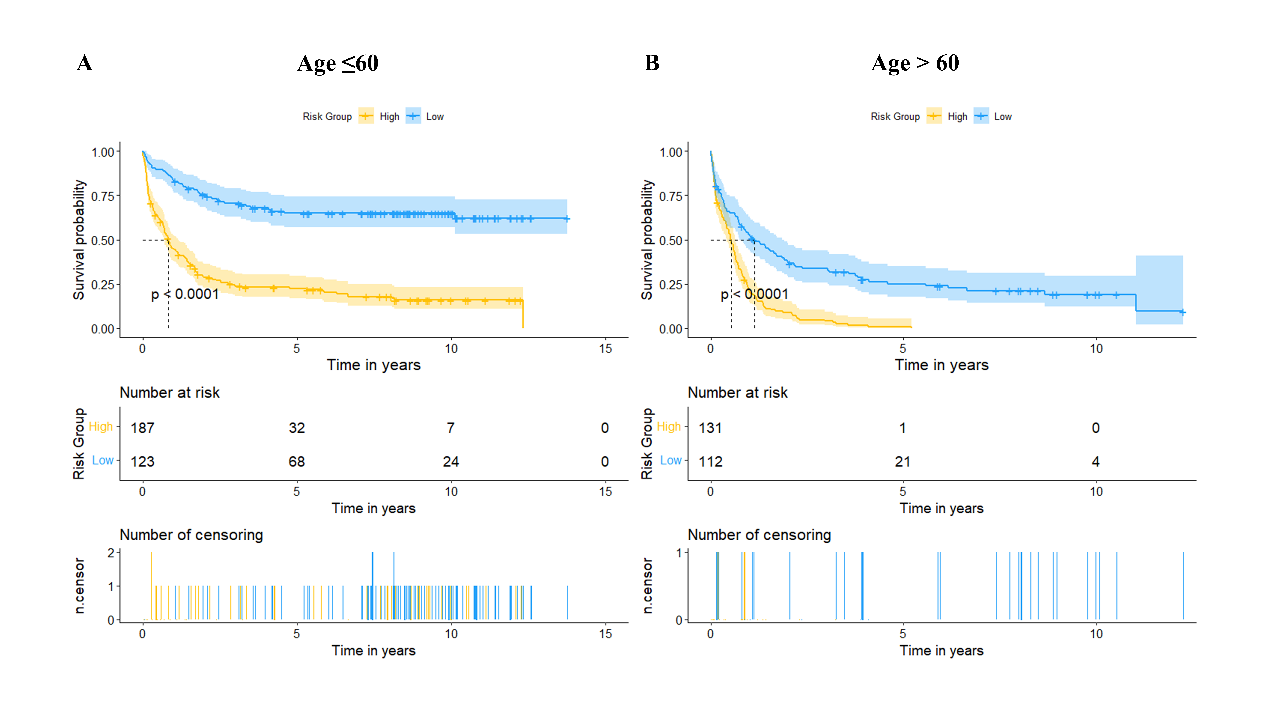


### Figure S4 The Kaplan-Meier plot of different age groups in GSE12417 dataset. (A) The Kaplan-Meier plot shows OS differences of those age ≤60 years according to risk score stratification. (B) The Kaplan-Meier plot shows OS differences of those age >60 years according to risk score stratification.


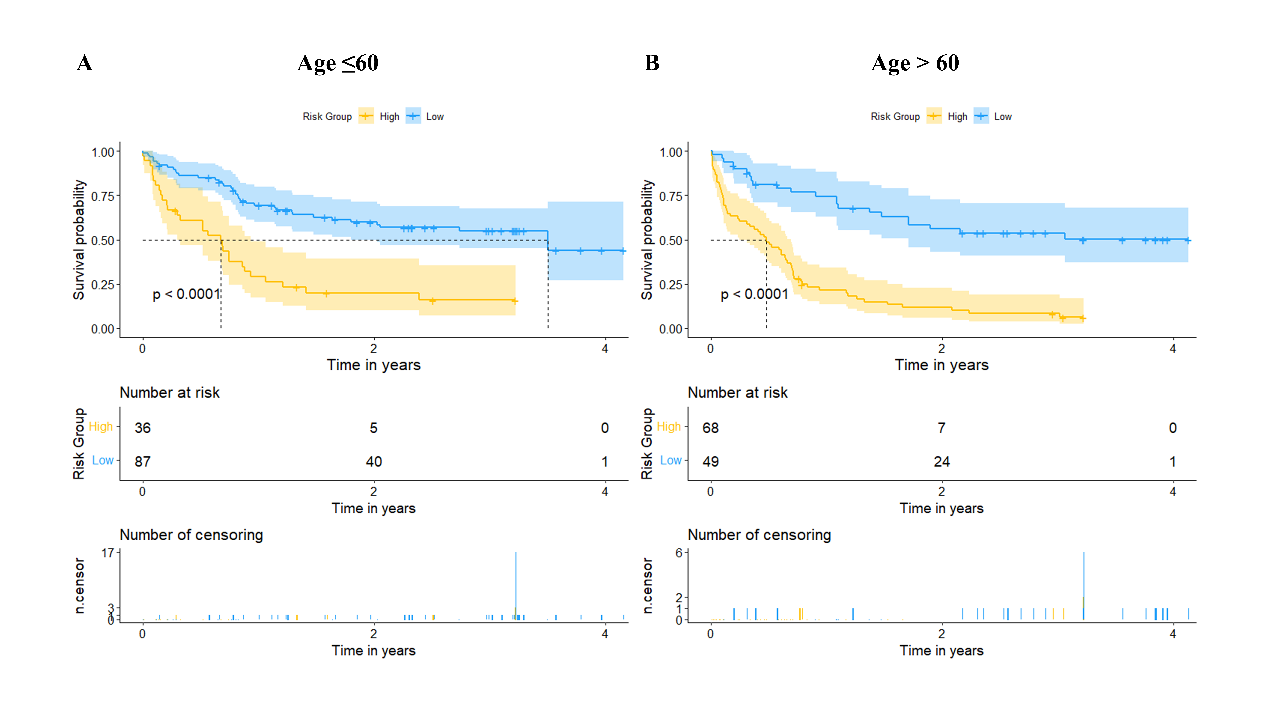


## Stacking model validation and comparisons for different machine learning

### Table S2 Setting of hyper-parameters for different machine learning.

| Machine learning algorithms | Hyper-parameter | Range | Step |
| --- | --- | --- | --- |
| RSF | Number of trees | 1000 (fixed) |  |
|  | sampsize | 0.63 (fixed) |  |
|  | nsplit | 10 (fixed) |  |
| Survival - SVM | gamma | 1 (fixed) |  |
| GBM | Number of trees | 10000 (fixed) |  |
|  | Depth of interaction | 2 (fixed) |  |
|  | Number of minobsinnode | 5 (fixed) |  |
|  | shrinkage | 0.005 (fixed) |  |
| XGBoost | max depth | 3~6 | 1 |
|  | eta | 0.01~0.1 | 0.01 |
|  | gamma | 0.1~0.5 | 0.1 |
|  | subsample | 0.8 (fixed) |  |
|  | colsample bytree | 1 (fixed) |  |
|  | min child weight | 5~20 | 5 |
|  | nround | 400 (fixed) |  |
|  | early_stopping_rounds | 25 (fixed) |  |

###
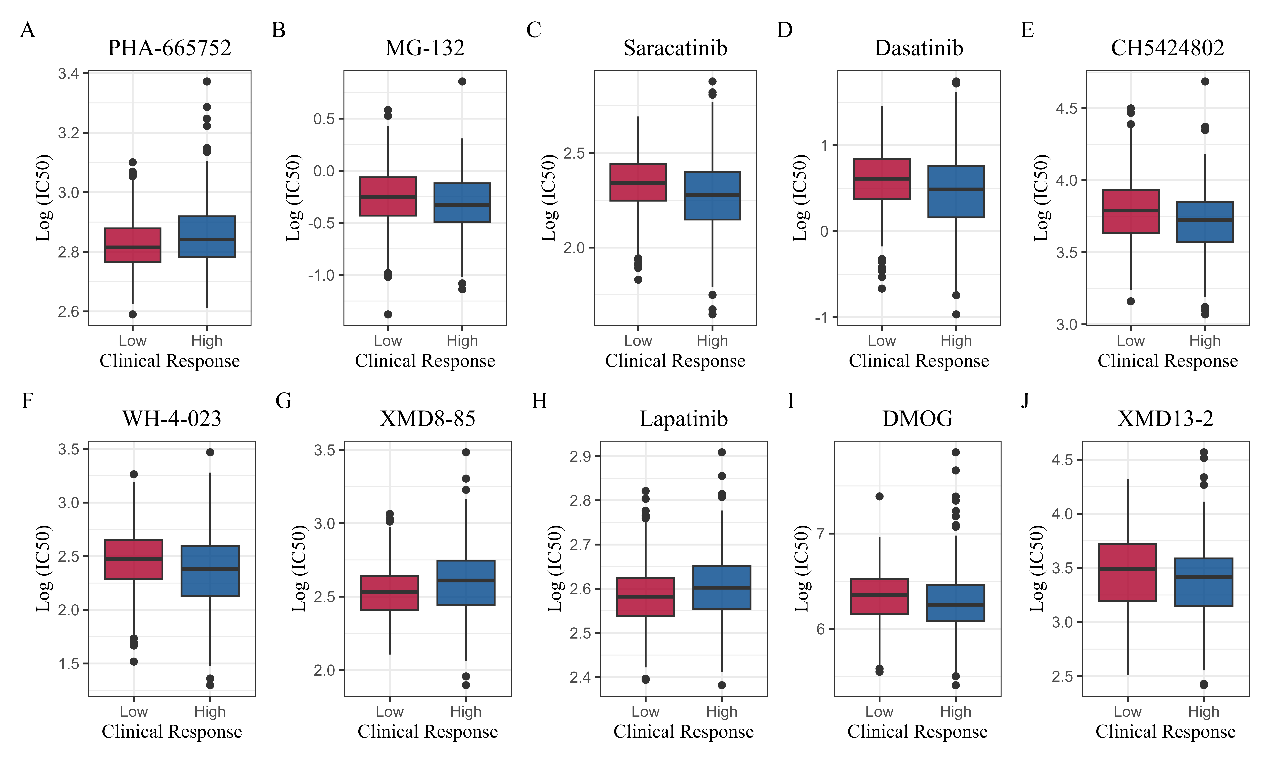
Figure S5 The discrepancies of top 10 drug sensitivity by p value between different risk groups. Smaller IC50 values indicate greater sensitivity to the drug.

IC50 = half-maximal inhibitory concentration.

### Figure S6 Bootstrap ROC for stacking model. Internal validation was carried out by a 1000-times bootstrap method.


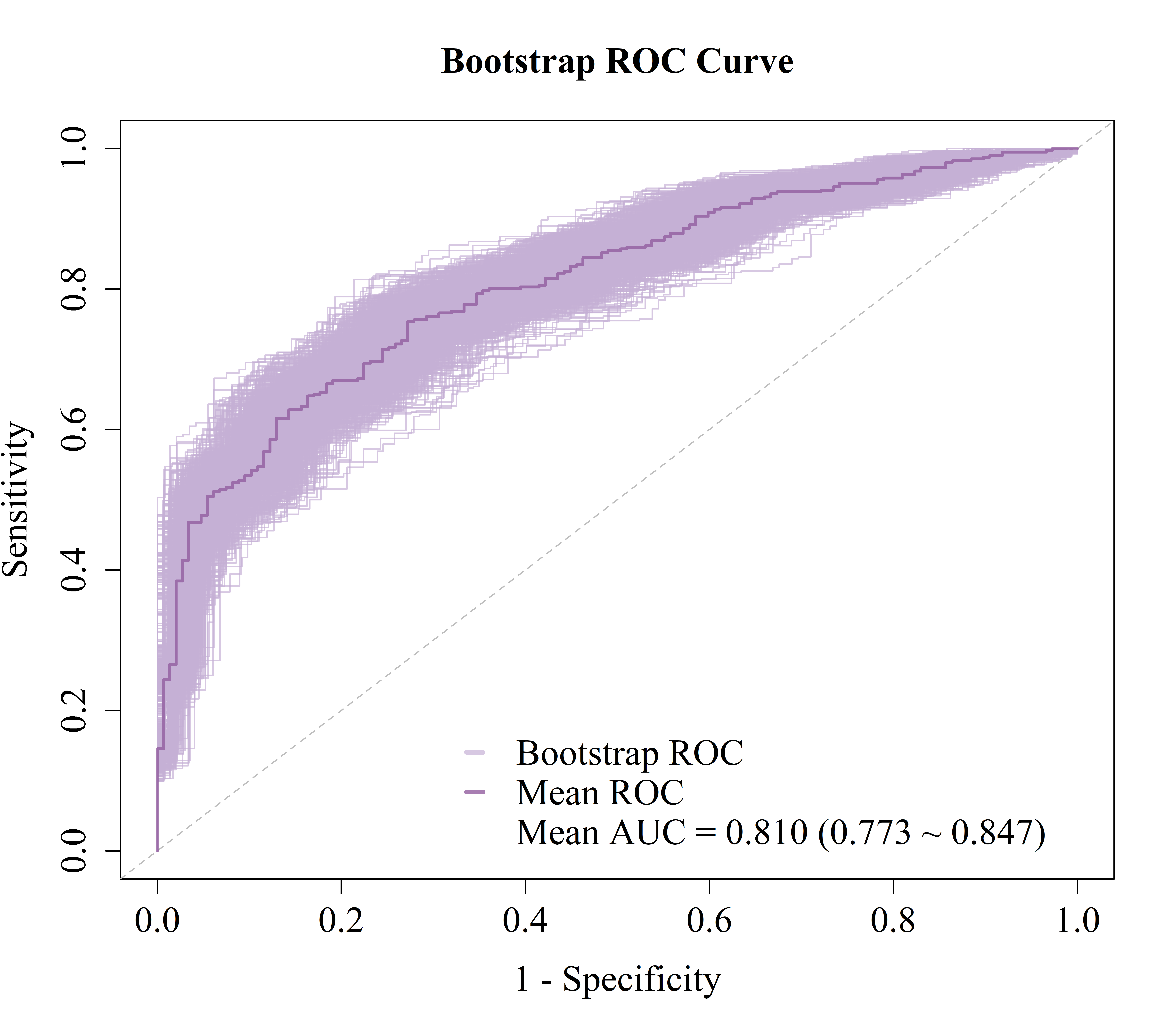


### Figure S7 Comparison of 1-,2-,3-year ROC curves for random survival forest model with different predictors in validation dataset (TCGA-LAML). (A) Random survival forest model with stacking linear predictor; (B) Random survival forest model simply incorporates all predictors.


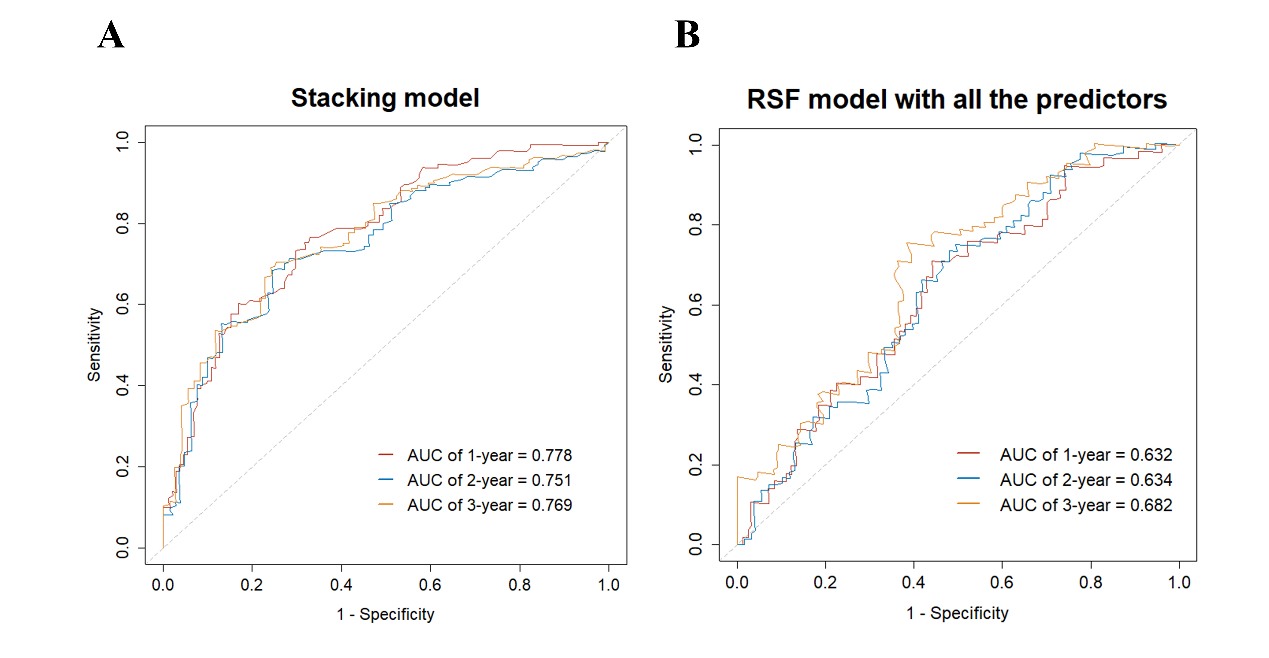


### Figure S8 Comparison of time dependent AUC among different machine learning.

**
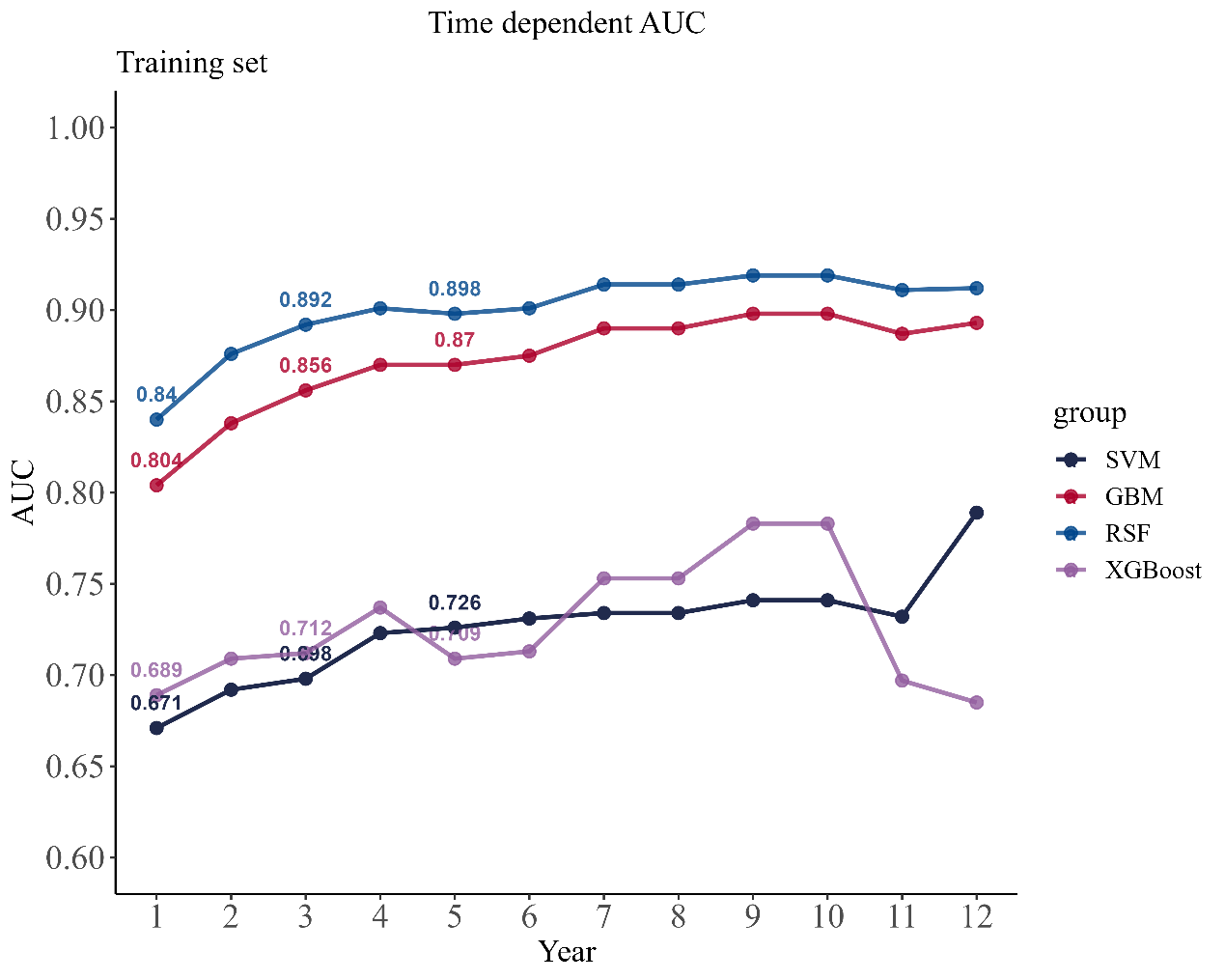
**

SVM = survival support vector machine;

GBM = generalized boosted regression modeling;

RSF = random survival forest;

XGBoost = eXtreme Gradient Boosting.

### Figure S9 Bootstrap ROC for RSF model with stacking linear predictor. Internal validation was carried out by a 1000-times bootstrap method.


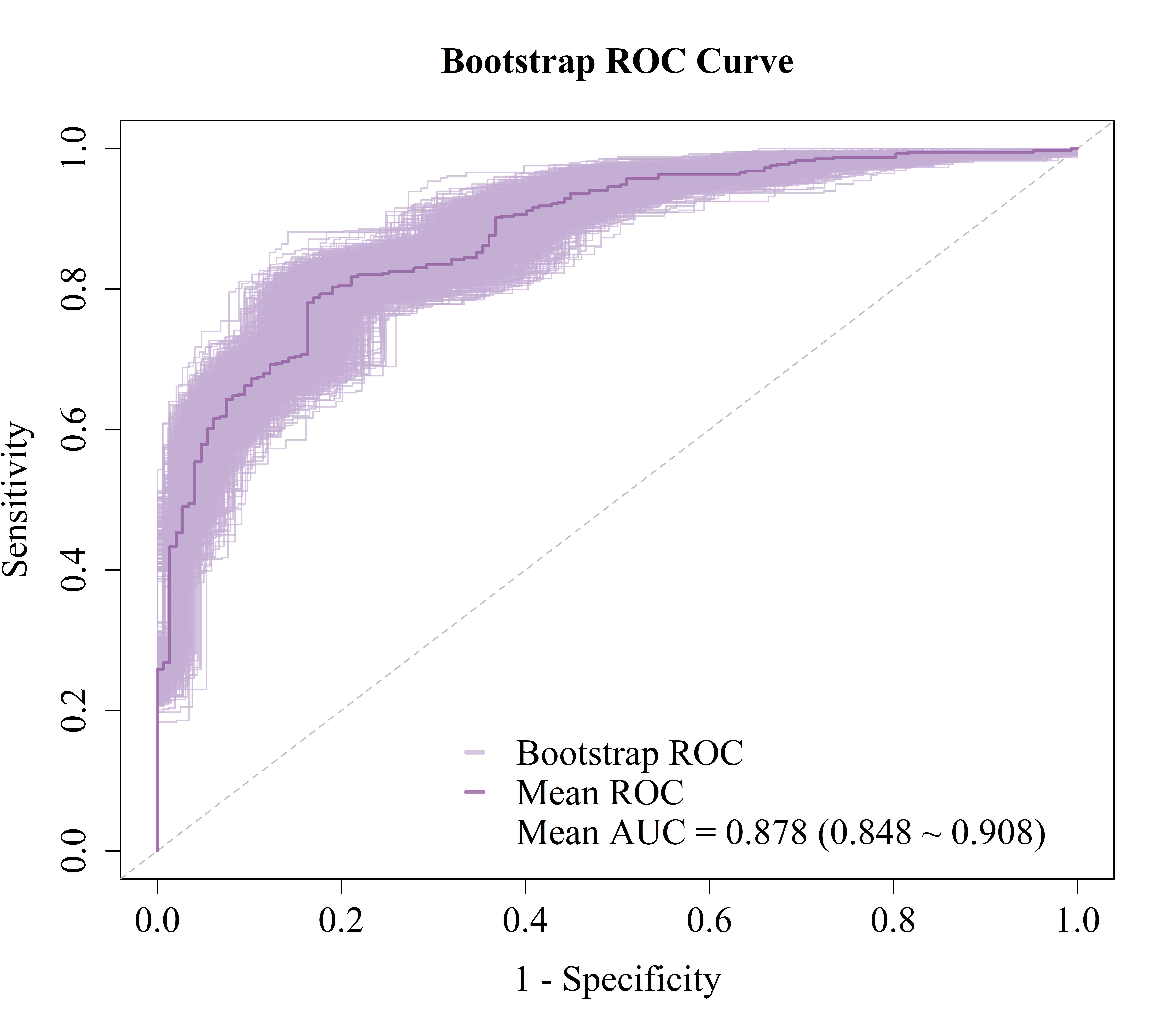


## Personalised Prediction

### Figure S10 Individual predictions for AML patients using rsf model with stacking linear predictor and clinical factors.


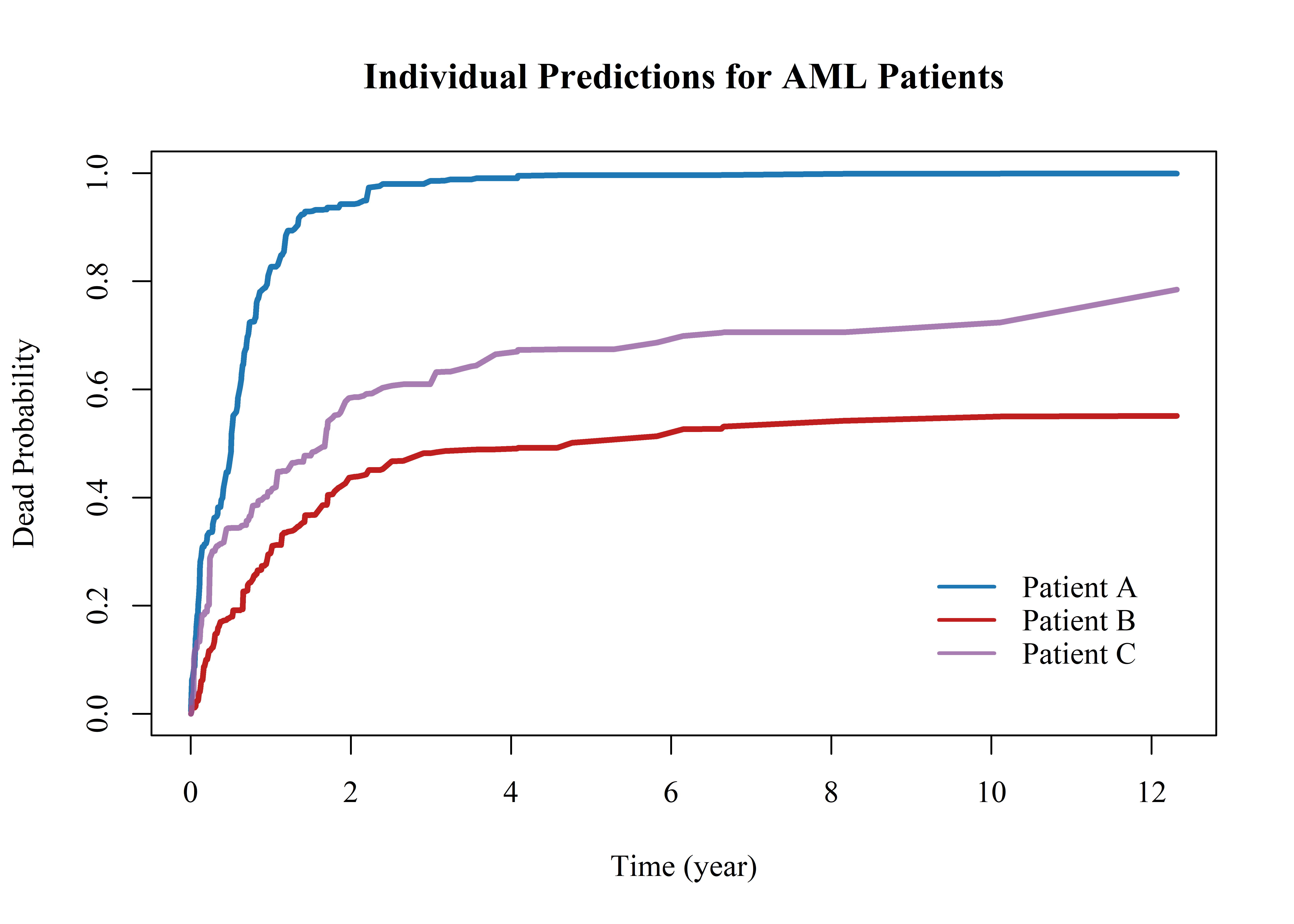

Supplement: Supplementary file 1 — Supplementary Information 1. [file 41598_2024_53306_MOESM1_ESM.docx]
